# Landscape Pattern and Plant Diversity in an Arid Inland River Basin: A Structural Equation Modeling Approach Based on Multi-Source Data

**DOI:** 10.3390/biology14081100

**Published:** 2025-08-21

**Authors:** Hui Shi, Tiange Shi

**Affiliations:** 1 College of Tourism, Xinjiang University of Finance and Economics, Urumqi 830012, China; shihui_129@163.com; 2 College of Economics, Xinjiang University of Finance and Economics, Urumqi 830012, China; 3 Xinjiang Institute of Ecology and Geography, Chinese Academy of Sciences, Urumqi 830011, China

**Keywords:** plant diversity, landscape pattern, landscape connectivity, PLS-SEM, Hotan River Basin

## Abstract

Biodiversity in arid river basins is highly climate-sensitive, yet the multi-pathway relations among the environment, landscape structure, connectivity, and plant diversity remain unclear. Framed by a scale–place–space sustainability perspective, we evaluated, in the Hotan River Basin (NW China), how the environmental factors affect plant diversity directly and indirectly via the landscape configuration and functional connectivity. We integrated Landsat images (2000, 2012, and 2023), 57 vegetation plots, topographic and meteorological data; computed the landscape indices and Conefor connectivity metrics (PC, IIC); and fitted a partial least squares structural equation model (PLS-SEM). From 2000 to 2023, the bare land declined, converted mainly into shrubland and cropland; the construction land is projected to expand under SSP1-2.6/SSP2-4.5/SSP5-8.5 by 2035 and 2050. The landscape metrics showed a rising PD, DIVISION, and SHDI/SHEI, and a declining AI and CONTAG, indicating finer, more heterogeneous mosaics. Plant diversity peaked on low–moderate slopes and with ~32–36 mm annual precipitation. The PLS-SEM revealed significant direct effects on diversity from environmental factors (positive), landscape structure (negative), and connectivity (positive). The dominant chained mediation (environment → structure → connectivity → diversity) indicated that environmental constraints first reconfigure the spatial structure and then propagate to community responses via connectivity, highlighting connectivity’s role in buffering climatic stress and stabilizing communities. The findings provide a quantitative framework to inform biodiversity conservation and sustainable landscape planning in arid basins.

## Simple Summary

Arid river basins are very sensitive to climate and land-use change, but it is unclear how the environment, landscape structure, and habitat connections together shape plant diversity. We studied the Hotan River Basin in northwest China using satellite images (2000, 2012, and 2023), field plots, terrain, and climate data. Over the past two decades, the bare land shrank and was mainly replaced by shrubland and cropland, while the construction land expanded. The landscape became more fragmented and heterogeneous (more, smaller, less-clumped patches). Plant diversity was highest on gentle-to-moderate slopes and where the annual rainfall was about 32–36 mm. Using a statistical pathway model, we found that the environmental conditions directly increase plant diversity, but the way the land is arranged (its structure) could reduce it when fragmentation is high. The habitat connectivity—how well patches are linked for seed and pollen movement—directly promotes diversity. The strongest pathway is the environment, which first reshapes the landscape structure, and then affects connectivity and ultimately plant diversity. These insights support “connectivity-first” planning in drylands: protect and restore the riparian and shrubland corridors and stepping-stone patches, guide construction away from the key links, and maintain environmental flows and shallow-groundwater belts to keep habitat networks functional under climate stress.

## 1. Introduction

Plant diversity, a key component of biodiversity, underpins ecosystem stability and human well-being [1,2,3]. However, global climate change, accelerating urbanization, and rapid land-use changes have led to extensive habitat loss and increased landscape fragmentation, posing serious threats to plant diversity [4,5]. In recent years, understanding the spatial patterns of plant species diversity and their driving mechanisms have become a central focus in biodiversity research [6]. Such knowledge is essential for elucidating community assembly and succession processes and provides a theoretical foundation for assessing ecosystem responses to environmental disturbances and their potential for recovery [7].

In recent decades, the impacts of global climate change have intensified, with arid and semi-arid ecosystems being particularly sensitive due to their chronic water limitations and limited buffering capacity [8]. Climate change acts as a primary driver influencing primary productivity, water availability, and community structure, and can reconfigure ecohydrological regimes and vegetation trajectories [9,10]. Broad-scale climatic variables, such as temperature and precipitation, shape species distribution and ecological strategies by altering habitat suitability and resource accessibility [11,12]. The topographic heterogeneity contributes to microclimatic diversity, with factors such as elevation and slope regulating solar radiation, temperature gradients, and soil moisture. These conditions create a variety of ecological niches, promoting species coexistence and increasing community structural complexity [13,14]. In parallel, human activities restructure landscape composition and configuration, intensifying habitat fragmentation, severing ecological corridors, and increasing dispersal resistance, thereby constraining species’ ranges and ultimately eroding regional plant biodiversity [15].

Landscape patterns, as spatial representations of ecosystem structure and function, are increasingly recognized in biodiversity research [16]. The metrics describing the patch number, shape, area, and spatial configuration capture the degree of heterogeneity and stability within ecosystems, directly influencing habitat accessibility, dispersal routes, and population persistence [17,18]. Under global environmental change, shifts in landscape structures have become the key drivers of plant community composition and ecosystem functioning [16,19]. The landscape connectivity, which reflects the spatial continuity between habitat patches, plays a critical role in maintaining ecological processes and stabilizing species dynamics [20,21]. It influences species distribution at the landscape scale and is shaped by matrix permeability, patch configuration, and species-specific traits [22,23]. Although growing research has linked connectivity to dispersal, migration, and genetic diversity, most studies still examine climate impacts on species distributions in isolation, lacking an integrated view with the landscape configuration and connectivity [9,24]. Empirical studies that integrate climate, landscape structure, and connectivity in a unified analytical framework remain limited [25,26]. Moreover, conventional approaches, such as generalized linear mixed models and redundancy analyses, struggle to capture the complex, nonlinear, and mediated pathways among the interacting variables—limitations especially evident in structurally complex arid ecosystems [1,5,27].

Partial least squares structural equation modeling (PLS-SEM) has emerged as a powerful tool for disentangling multifactor causal mechanisms [28,29]. It effectively handles non-normal data, multicollinearity, and small samples, enabling the simultaneous estimation of direct, indirect, and mediating effects among the latent constructs [30]. PLS-SEM has been successfully applied to model ecological interactions in highly variable environments, particularly in drylands where the theoretical frameworks are often uncertain. Previous studies have used this method to reveal how climate, land use, and connectivity jointly affect biodiversity across forests, bird communities, and arid vegetation systems, highlighting its theoretical adaptability and empirical strength [31,32].

This study focuses on the Hotan River Basin, a typical arid inland river catchment in the southwestern Tarim Basin, China. The region features a distinct desert–oasis transition zone with important ecological functions and biodiversity value [33]. Over the past two decades, hydrological interventions, such as the Tarim River regulation project and “water transfer from north to south”, have significantly altered the local hydrology and landscape structure, with pronounced effects on the patch configuration, connectivity, and biodiversity. Against this backdrop, based on the theory of environmental sustainability, from the perspective of scale–place–space, our goal was to evaluate how environmental factors influence plant diversity directly and indirectly via the landscape structure and connectivity, and to place these pathways in a multi-scale, place-based sustainability context. Using multi-temporal remote sensing data from 2000, 2012, and 2023, together with field vegetation plots, DEM-derived terrain metrics, and climate data, we constructed a PLS-SEM model to explore the interactions among the environmental factors, landscape patterns, connectivity, and plant diversity. The specific objectives were as follows: (1) characterize the landscape pattern changes across the three time periods and, using the Patch-generating Land Use Simulation (PLUS) model, project the land cover for 2035 and 2050 under SSP1-2.6, SSP2-4.5, and SSP5-8.5; (2) analyze spatial patterns of plant diversity along hydro-topographic gradients; and (3) identify the causal pathways linking the environmental and spatial factors to plant diversity. By embedding dryland biodiversity within an environment → structure → function → diversity causal chain, the study delivers pathway-based, connectivity-informed evidence to guide climate-robust corridor design, water–land coordination, and conservation prioritization in arid river basins, thereby advancing a scalable (scale–place–space) framework for environmental sustainability.

## 2. Materials and Methods

### 2.1. Study Area

The Hotan River Basin is located in the southwestern Tarim Basin, Xinjiang, China, within an extremely arid region. It spans 36°45′7″ to 40°35′50″ N and 77°22′5″ to 81°33′21″ E, covering approximately 28,644 km^2^ and including eight administrative divisions, such as Hotan City and Aksu City. The basin is bounded by the Kunlun and Karakoram Mountains to the south and the Taklimakan Desert to the north, with the terrain sloping from south to north.

The basin comprises two major tributaries: the Yulongkashi River and the Karakashi River, which originate from high mountain snowmelt and converge to form the Hotan River. The region has a warm temperate continental arid climate, with average annual temperatures of 10.5–12.8 °C, precipitation of 35–55 mm, and evaporation exceeding 2500 mm. The seasonal runoff variation is pronounced, with peak flows in summer and low flows in winter.

**Figure 1:**
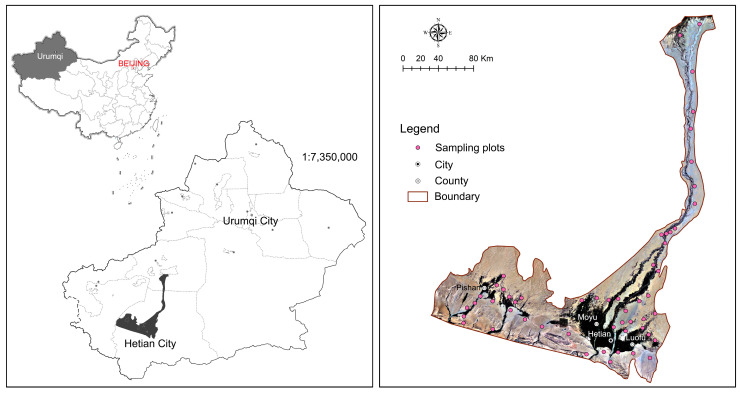
The location of the study area and sampling points.

Ecologically, the Hotan River Basin supports a fragile yet functionally critical desert riparian ecosystem, shaped by strong hydrological dependence and sharp environmental gradients. It serves as a key habitat for drought-adapted and relict species, such as *Populus euphratica* and *Tamarix* spp., as well as numerous desert herbs and shrubs. The complex interplay between limited water availability, elevation gradients, and land-use changes has resulted in high landscape heterogeneity and ecological sensitivity. These features endow the region with a high conservation priority and make it a representative natural laboratory for studying the impacts of environmental and landscape dynamics on plant diversity in arid inland river basins (Figure 1).

### 2.2. Data Sources and Processing

#### 2.2.1. Remote Sensing Data and Processing

To analyze the landscape pattern dynamics in the Hotan River Basin, Landsat satellite imagery was obtained from the USGS Earth Explorer platform (http://earthexplorer.usgs.gov/). Three images from the growing seasons (May–August) of 2000, 2012, and 2023 were selected, with a spatial resolution of 30 m and cloud cover below 5%, ensuring data quality and comparability. The elevation data (DEM) were sourced from the Geospatial Data Cloud (http://www.gscloud.cn). A national 1:1,000,000 vegetation map was acquired from the Peking University Geographic Data Platform. The base vector layers—including roads, rivers, settlements, and administrative boundaries—were sourced from the National Catalogue Service for Geographic Information (NCSGI) (http://www.webmap.cn). Gridded datasets for the annual precipitation, mean annual temperature, population, GDP, and soil types at 1 km resolution were obtained from the Resource and Environment Science and Data Center, Chinese Academy of Sciences (RESDC) (resdc.cn). Scenario-based land-use inputs were taken from Land-Use Harmonization v2 (LUH2) (http://www.luh.umd.edu) (Table 1).

All imagery was preprocessed in ENVI 5.3 and ArcGIS 10.8, including radiometric calibration, atmospheric correction, and geometric correction. Based on spectral and textural features, we performed object-based classification using nearest-neighbor classifier, complemented by expert visual interpretation, to map landscape types for 2000, 2012, and 2023. Classification outputs were validated and iteratively refined against high-resolution Google Earth imagery and 2023 field plots, yielding overall accuracies > 89% for each year.

Mapped classes were reclassified following Land Use/Cover Classification System (GB/T 21010—2017) and adapted to local ecological characteristics [34]. Five final categories were defined: construction land, wetland, cultivated land, bare land, and shrubland.

For subsequent PLUS simulations and PLS-SEM analyses, all raster and vector inputs were resampled to common spatial grid and projected to WGS_1984_UTM_Zone_44N to ensure spatial consistency across datasets.

#### 2.2.2. Vegetation Plot Data

The vegetation data were obtained from field surveys conducted during the peak growing seasons (July) of 2012 and 2023 in representative areas of the Hotan River Basin. The survey sites extended longitudinally along the river valley from north to south, covering the confluence zone of the Yulongkashi and Karakashi Rivers and adjacent ecological transition zones (Figure 1).

A total of 57 permanent plots were established using a typical sampling method, with each plot measuring 10 m × 10 m and three replicates per site. Within each plot, five nested 1 m × 1 m quadrats were set for detailed herbaceous vegetation assessment. To capture the vegetation variation along moisture and disturbance gradients, additional 1 m × 1 m quadrats were established at distances of 30 m to 1710 m from the riverbank and 60 m to 360 m from roadsides. Each quadrat was surveyed for the plant species’ names, cover, abundance, height, and crown width. The environmental variables recorded at each plot included the latitude, longitude, elevation, slope, aspect, soil type, and depth to groundwater table, following standard protocols [16,35].

### 2.3. Research Methods

#### 2.3.1. Simulation and Prediction of the Landscape Types Under Different Scenarios of Climate Change

- PLUS Model

We simulated the future land-cover dynamics with the Patch-generating Land Use Simulation (PLUS) model, which couples a random forest-based Land Expansion Analysis Strategy (LEAS) with a multi-type random seed Cellular Automata (CARS) engine. LEAS learns the relationships between the expansion of each land-cover class and a set of biophysical and socioeconomic drivers, producing gridded development probability surfaces and the variable importance for each class. CARS then allocates the patches using a decreasing-threshold, multi-type seed mechanism that ensures a realistic patch geometry and contagion. This hybrid design has been shown to explain land-use change mechanisms and to reproduce patch evolution under alternative scenarios [36].

Guided by regional conditions and prior studies, we used ten drivers covering environment and human activity: mean annual temperature, mean annual precipitation, elevation, slope, soil type, distance to rivers, population density, GDP, distance to settlements, and distance to roads (all rasters resampled to match analysis grid) [37].

Using 2000 and 2012 land-cover maps as inputs, LEAS estimated class-specific development probabilities; CARS then simulated 2023 land cover from historical trend. Simulated 2023 map was validated against interpreted 2023 classification, yielding overall accuracy = 91.83% and Kappa = 0.7703, indicating that PLUS reliably reproduced observed landscape patterns in basin. Calibrated model was subsequently used to project 2035 and 2050 land cover.

All simulations used five reclassified types: construction land, wetland, cultivated land, bare land, and shrubland.

- SSP–RCP Scenario Design

To represent plausible futures, we combined Shared Socioeconomic Pathways (SSPs) with Representative Concentration Pathways (RCPs) and defined three scenarios.

SSP1-2.6 (sustainability, low forcing): Prioritize ecological benefits. Conversion probabilities from cultivated land/shrubland to construction land were reduced by 30% and wetland → construction land by 20%; that for construction land → vegetation increased by 10%. Lakes/reservoirs were constrained as non-convertible to preserve aquatic systems.

SSP2-4.5 (natural growth, medium forcing): Medium forcing and continuation of recent trends. No additional conversion penalties were imposed beyond transition matrix derived from 2000 to 2023; apart from construction land and open water constraints, classes could mutually convert following learned probabilities.

SSP5-8.5 (economic development, high forcing): High forcing with development priority. Based on SSP2-4.5, probability of other classes → construction land was increased by 20%, while probability of construction land → other classes was reduced by 30%, reflecting stronger development pressure.

Scenario rules were operationalized via class-specific conversion cost matrix and calibrated neighborhood (domain) weights within CARS, consistent with established practice for patch-level allocation; neighborhood weights and transition matrix were parameterized with reference to prior studies and tuned to reproduce observed patch geometry and contagion [36,37].

- Land-Demand Alignment under SSP–RCP

We derived the class-level area demands for 2035 and 2050 from the LUH2 (CMIP6) land-use harmonization dataset and reconciled them with the basin’s 2023 baseline using a simple scaling factor [38]:

(1) $${Area}_{local,i,t}={Area}_{LUH2,i,t}\times K_{i},\;K_{i}=\frac{{Area}_{local,i,2023}}{{Area}_{LUH2,i,2023}}$$

where *i* is the land-cover class and *t* ∈ {2035, 2050}. The scaled demands ${Area}_{local,i,t}$ are used as global constraints in PLUS so that the allocated patches match both (i) the scenario storyline (via the conversion rules) and (ii) the basin-specific baseline (via $K_{i}$). This ensures internally consistent projections across the construction land, wetland, cultivated land, bare land, and shrubland.

#### 2.3.2. Landscape Pattern Index Calculation

Landscape metrics are widely used to quantify the spatial composition and configuration, providing an effective means to assess landscape heterogeneity and temporal changes [39]. In this study, we used Fragstats 4.2 to calculate the landscape metrics at both the class and landscape levels for the years 2000, 2012, and 2023, based on the ecological characteristics of the Hotan River Basin.

At the landscape level, the metrics were selected based on autocorrelation and multicollinearity tests. The final set included the patch density (PD), Aggregation Index (AI), Shannon’s diversity index (SHDI), Shannon’s evenness index (SHEI), Contagion Index (CONTAG), Interspersion and Juxtaposition Index (IJI), and landscape division index (DIVISION). At the class level, five indices were selected: Number of Patches (NP), PD, largest patch index (LPI), landscape shape index (LSI), and IJI [40,41].

For the subsequent structural equation modeling, a 4 km × 4 km grid was used as the basic analysis unit to ensure the applicability of landscape variables at the local scale. Spatial matching was performed between the grids and vegetation sampling plots. The landscape metrics were recalculated at the grid scale and re-evaluated for spatial autocorrelation and multicollinearity. Based on this, six representative landscape-level metrics were retained: edge density (ED), LSI, SHDI, SHEI, Simpson’s Diversity Index (SIDI), and Simpson’s Evenness Index (SIEI).

#### 2.3.3. Landscape Connectivity Calculation

Landscape connectivity refers to the degree to which a landscape’s structure facilitates or impedes the movement of organisms. It includes two components: the structural connectivity, which describes the geometric relationships among the habitat patches (e.g., distances and shapes), and the functional connectivity, which incorporates species’ dispersal abilities and behavioral responses and the landscape attributes, offering greater explanatory power for ecological processes [20,42].

In the Hotan River Basin, riparian brushlands represent the key habitats for native desert species and serve as the primary corridors for ecological flow. To ensure the ecological relevance of connectivity analysis, brushland patches larger than 1 km^2^ were identified as source patches. Adjacent smaller patches were merged to construct a base landscape network capable of reflecting ecological processes.

Based on this source patch network, we used ArcGIS 10.8 and Conefor 26 to calculate the landscape connectivity. Two widely used structural indices were selected: the integral index of connectivity (IIC) and the probability of connectivity (PC), with the following formulas:

(2) $$IIC=\frac{\sum_{i=1}^{n}\sum_{j=1}^{n}\frac{a_{i}\;\cdot \;a_{j}}{1+nl_{ij}}}{A_{L}^{2}}$$

(3) $$PC=\frac{\sum_{i=1}^{n}\sum_{j=1}^{n}a_{i}\;\cdot \;a_{j}\;\cdot \;P_{ij}^{*}}{A_{L}^{2}}$$

where *a*_*i*_ and *a*_*j*_ are the areas of patches *i* and *j*, *nl*_*ij*_ is the number of links in the shortest path between patches, *A*_*L*_ is the total landscape area, and *P*_*ij*_^∗^ is the maximum product probability of all paths between patches.

Seventeen threshold distances were tested (50 m to 20 km) to simulate different species dispersal ranges. The IIC and PC increased with distance, reaching stability at 10 km. Thus, 10 km was identified as the functional connectivity threshold, and corresponding IIC and PC values were used as the observed indicators for the latent variable “connectivity” in the structural equation model (Table 2).

#### 2.3.4. Plant Community Diversity Assessment

To evaluate plant community diversity, four indices were calculated from 57 field plots: importance value (IV), Simpson’s Dominance Index (D), Shannon–Wiener diversity index (H′), and Pielou’s evenness index (J). IV integrates relative cover, density, and height. D reflects species dominance and distribution evenness (lower D indicates higher evenness). H′ accounts for species richness and evenness. J evaluates evenness relative to species number [35,43].

Formulas are as follows:

(4) $$IV=\frac{Rel.Cover+Rel.Density+Rel.Height}{3}$$

(5) $$D=1-\sum P_{i}^{2}$$

(6) $$H^{\prime }=-\sum P_{i}{lnP}_{i}$$

(7) $$J=H^{\prime }/lnS$$

(8) $$P_{i}=N_{i}/N$$

where *S* is species richness, *N*_*i*_ is number of individuals of species *i* in sample plot, and N is total number of individuals of all species in sample plot.

#### 2.3.5. PLS-SEM Construction

To investigate the causal pathways between the environmental factors, landscape patterns, connectivity, and plant diversity, a PLS-SEM was developed using SmartPLS 4.0. A nearest distance matching method was applied to spatially align the remote sensing data, landscape indices, and field data at the grid level.

Four latent variables were defined: environmental factors (ENV), landscape pattern (LSP), connectivity (CON), and plant diversity (DIV). The indicator pools drew on the study area diagnostics, the driver set adopted in the PLUS model, and the prior literature, then were screened through iterative PLS-SEM estimation in SmartPLS 4.0. The indicators were retained only when the standardized loadings were ≥0.50 and the construct reliability/validity was acceptable; all the spatial predictors were harmonized onto a 4 × 4 km grid:

- The ENV comprised the distance to settlements (D_set), soil type (st), groundwater depth (gwd), precipitation (prcp), and slope [44];
- The LSP was operationalized with seven grid-based metrics computed from the 2023 classification: the edge density (ED), patch density (PD), landscape shape index (LSI), largest patch index (LPI), Shannon’s diversity (SHDI), Shannon’s evenness (SHEI), and landscape division index (DIVISION) [31,45];
- The CON was quantified by the integral index of connectivity (IIC) and the probability of connectivity (PC), evaluated at a 10 km dispersal threshold derived from the source-patch network [46];
- The DIV was operationalized from the field plots using four standard indices: the IV, D, H′, and J [16].

**Figure 2:**
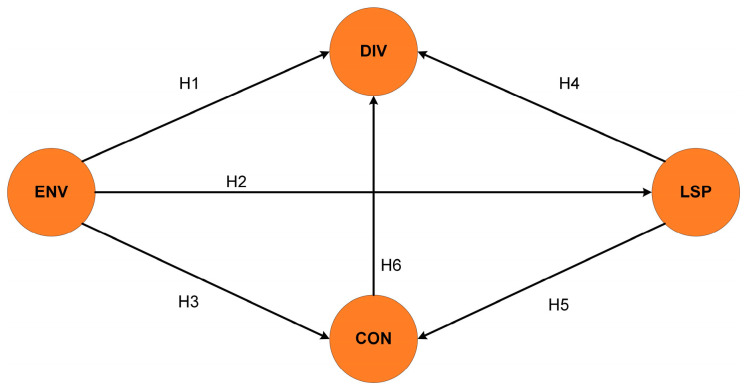
PLS-SEM conceptual model diagram.

Drawing from ecological niche theory, landscape–structure–function theory, and island biogeography, the following hypotheses were proposed (Figure 2):

1. Direct Effects

H1: ENV → DIV

H2: ENV → LSP

H3: ENV → CON

H4: LSP → CON

H5: LSP → DIV

H6: CON → DIV

2. Mediated and Chain Effects

H7: ENV → CON → DIV

H8: LSP → CON → DIV

H9: ENV → LSP → DIV

H10: ENV → LSP → CON

H11: ENV → LSP → CON → DIV (chain mediated)

The model was estimated using the PLS algorithm with 5000 bootstrap samples (two-tailed tests, α = 0.05; 95% confidence). The model quality was assessed following established guidelines [28,47,48]:

- Reliability: Cronbach’s alpha and composite reliability (>0.7).
- Convergent validity: Average variance extracted (AVE > 0.5) and standardized indicator loadings (ideally > 0.70; minimum acceptable > 0.50).
- Discriminant validity: Fornell–Larcker criterion (square root of AVE exceeds inter-construct correlations) and HTMT with 95% confidence intervals not including 1.
- Model fit: Standardized root mean square residual (SRMR < 0.1).
- Explanatory/predictive power: R^2^ for endogenous constructs; effect sizes f^2^ interpreted per Cohen’s benchmarks (~0.02 small, ~0.15 medium, and ~0.35 large); Stone–Geisser Q^2^ > 0 (blindfolding).
- Path inference: Significance of path coefficients evaluated via bootstrapped t-statistics and *p*-values.

## 3. Results

### 3.1. Analysis of Landscape Pattern Evolution

#### 3.1.1. Dynamic Area Changes in the Landscape

**Figure 3:**
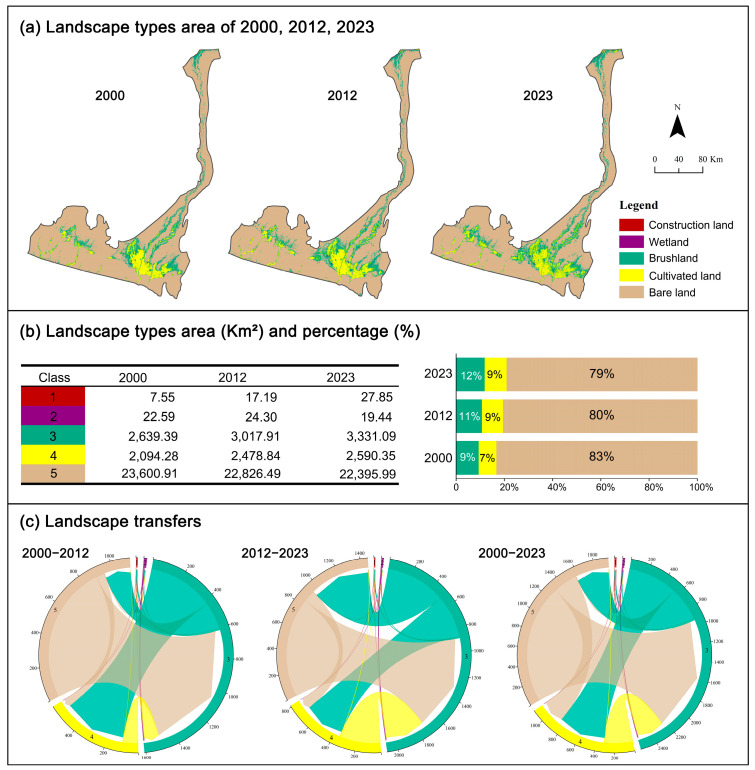
Landscape type areas and transfers of area in 2000, 2012, and 2023.

Based on the remote sensing classification results from 2000, 2012, and 2023 (Figure 3), the landscape of the Hotan River Basin was dominated by bare land, accounting for approximately 83% of the total area, followed by brushland. Between 2000 and 2023, the areas of construction land, brushland, and cultivated land increased by 20.3 km^2^, 691.7 km^2^, and 496.07 km^2^, respectively. In contrast, the area of bare land decreased significantly, with a cumulative reduction of 1204.92 km^2^, particularly between 2000 and 2012, when it declined by nearly 3%.

Significant spatial conversions occurred among the landscape types, particularly the transformation of bare land into other landscape types. Between 2000 and 2012, bare land was primarily converted into brushland (887.97 km^2^) and cultivated land (53.79 km^2^). Some brushland was also converted into cultivated land (429.82 km^2^) and construction land (8.10 km^2^). Between 2012 and 2023, the conversion of bare land continued, with 843.84 km^2^ transformed into brushland and 98.49 km^2^ into cultivated land. Meanwhile, the conversion of brushland into cultivated land declined to 362.34 km^2^. Over the entire study period (2000–2023), bare land was cumulatively converted into 1309.09 km^2^ of brushland and 263.48 km^2^ of cultivated land. The expansion of construction land was mainly at the expense of brushland, which contributed 16.03 km^2^ to its growth.

#### 3.1.2. Projected Land-Cover Change Under Climate–Socioeconomic Scenarios

**Figure 4:**
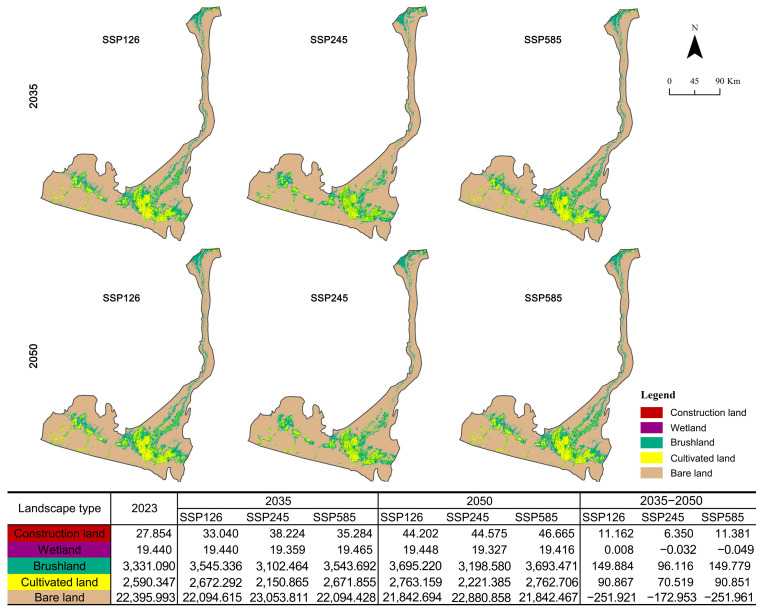
Areas of landscape types in Hotan River Basin in 2035 and 2050 under different scenarios.

Using the PLUS model, the land-cover was projected for 2035 and 2050 under three SSP–RCP scenarios: SSP1-2.6 (sustainability), SSP2-4.5 (natural growth), and SSP5-8.5 (economic development) (Figure 4). Across all scenarios and years, the Hotan River Basin remains dominated by bare land and shrubland. Relative to the 2023 baseline, the construction land consistently expands, the wetland shows only minor variations, and the cultivated land and shrubland exhibit scenario-dependent changes.

- SSP126 (sustainability): The bare land declines steadily (−301.379 km^2^ by 2035; −553.300 km^2^ by 2050), while all the other classes increase. The shrubland shows the largest absolute gains (+214.246 km^2^ in 2035; +364.131 km^2^ in 2050), followed by the cultivated land (+81.946 km^2^; +172.813 km^2^). The construction land exhibits the greatest proportional increase, rising 58.69% by 2050.
- SSP245 (middle of the road): The construction land continues to expand (+37.23% in 2035; +60.03% in 2050). The wetland area decreases slightly. The shrubland and cultivated land first contract and then recover, yet their 2050 areas remain below their 2023 levels. The bare land varies the most, peaking in 2035 (+657.818 km^2^) and then declining by 2050, but still exceeding the 2023 baseline.
- SSP585 (fossil-fueled development): The construction land, shrubland, and cultivated land all increase; the construction land shows the highest proportional growth (+26.67% in 2035; +67.53% in 2050) and reaches its largest extent among all the scenarios by 2050. The cultivated land also increased (+81.508 km^2^ in 2035; +172.359 km^2^ in 2050). The bare land persistently decreases (−301.565 km^2^ in 2035; −553.526 km^2^ in 2050), reaching its minimum by 2050.

#### 3.1.3. Changes in the Landscape Pattern Index

From 2000 to 2023, several key landscape pattern indices in the Hotan River Basin showed significant changes, indicating dynamic shifts in the spatial structure and ecological function. At the landscape level, the Number of Patches (NP), Shannon’s diversity index (SHDI), and Shannon’s evenness index (SHEI) all exhibited upward trends. The increase in the NP reflects a greater number of landscape patches per unit area, suggesting intensified fragmentation. SHDI increased from 0.4888 to 0.5022, and SHEI rose from 0.2728 to 0.2803, indicating increasing diversity in landscape types and a more balanced patch distribution—both signs of enhanced heterogeneity.

In contrast, the Aggregation Index (AI) and Contagion Index (CONTAG) showed a decreasing trend. The AI dropped from 99.6539 to 99.5765, suggesting reduced spatial clustering among similar patch types. The CONTAG declined by 0.4529, reflecting a growing proportion of small-scale patches and decreased spatial continuity of dominant landscape types, both indicative of increased fragmentation.

Meanwhile, the Interspersion and Juxtaposition Index (IJI) and landscape division index (DIVISION) demonstrated rising trends. The IJI values remained within the 35–40 range, indicating overall heterogeneity at a low-to-moderate level, but showing signs of gradual increase. The DIVISION increased from 0.2942 to 0.3508, reflecting a higher degree of landscape subdivision—consistent with the rising NP and the observed intensification of fragmentation (Table 3).

From 2000 to 2023, most land-use types—except bare land—exhibited increasing trends in patch density (PD), particularly for cultivated land, bushland, and construction land. The largest patch index (LPI) remained highest for bare land, followed by plowland and bushland, indicating that bare land continued to dominate in patch size despite a declining overall area.

In terms of shape complexity, bushland consistently exhibited the highest landscape shape index (LSI), followed by plowland, suggesting more irregular and fragmented patch boundaries in these vegetation-dominated landscapes.

The Interspersion and Juxtaposition Index (IJI) varied across landscape types. The reservoirs maintained IJI values between 50 and 60, showing a decreasing trend followed by recovery. The bushland and construction land, however, showed substantial increases in the IJI—from 45.17 to 51.91 and from 40.69 to 47.31, respectively—indicating rising heterogeneity and more complex spatial arrangements of patch types.

**Figure 5:**
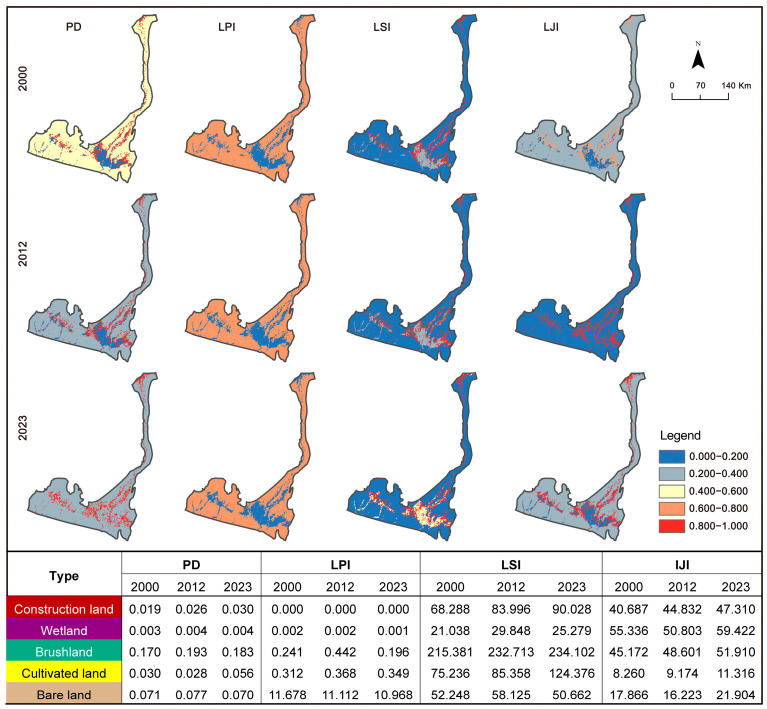
An index of landscape type scales in the Hotan River Basin in 2000, 2012, and 2023.

In summary, the landscape patterns in the Hotan River Basin from 2000 to 2023 showed increasing fragmentation, enhanced diversity, and greater structural complexity. These changes reflect a regional shift toward more heterogeneous spatial configurations (Figure 5).

### 3.2. Plant Community Characteristics

#### 3.2.1. Composition of Plant Communities

To better understand the vegetation composition of the Hotan River Basin, a total of 57 field plots were surveyed during the growing seasons of 2012 and 2023 across typical locations. Due to the region’s limited accessibility and extreme arid conditions, the plots were distributed along both riverbanks, the oasis margins, and the piedmont–desert transition zones. A total of 21 herbaceous species were recorded, belonging to 20 genera across 12 families. The dominant families included Chenopodiaceae (four genera, four species), Fabaceae (four genera, four species), Tamaricaceae (two genera, two species), Asteraceae (two genera, two species), and Salicaceae (one genus, two species). Other families, such as Solanaceae, Poaceae, Polygonaceae, Zygophyllaceae, Asclepiadaceae, Apocynaceae, and Amaranthaceae, were represented by one genus and one species each.

Based on a GIS spatial analysis and natural break classification, the study area was divided into three slope gradient zones—low (L: 0–4°), medium (M: 4–11°), and high (H: 11–71°)—to assess the impact of slope on the community composition.

(i) The vegetation exhibited a typical river-to-desert transition, with the life-form gradients ranging from trees (*Populus pruinosa*, *Populus euphratica*) to shrubs (*Tamarix chinensis*, *Reaumuria soongorica*) and perennial herbs (*Phragmites australis*, *Sophora alopecuroides*). Vegetation was mainly concentrated in the L and M zones, where the communities were more structurally diverse. In contrast, the vegetation in the H zone was sparse and the species composition was limited.
(ii) In the L zone, 16 species from 10 families were recorded. The dominant species with high importance values (IVs) included *R. soongorica*, *Seriphidium korovinii*, *P*. *australis*, *P*. *pruinosa*, and *P. euphratica*. The M zone also recorded 16 species, mainly from Amaranthaceae, Tamaricaceae, Salicaceae, and Polygonaceae. The dominant species included *Sympegma regelii*, *P*. *euphratica*, *Calligonum roborowskii*, and *T. chinensis*, indicating a transition zone characterized by residual trees and emerging shrub–herb communities. Only four species were identified in the H zone, with *P. euphratica* and *T. chinensis* dominating due to their high IVs.
(iii) The endemic species *C. roborowskii* and *S. korovinii* were primarily distributed in the M and parts of the L zones, representing key indicator species of the Kunlun–Tarim ecotone and reflecting ongoing regional aridification and desertification (Table 4).

#### 3.2.2. Plant Community Diversity

To explore the effects of precipitation gradients on plant diversity, a spatial analysis of long-term average precipitation was conducted using natural breaks classification. The study area was divided into five zones: <25 mm, 25–28 mm, 28–32 mm, 32–36 mm, and >36 mm. Combined with the slope-zone divisions (L, M, and H), the changes in plant diversity under different hydrothermal combinations were assessed (Table 5).

- L zone: Vegetation occurred across all five precipitation zones. The Shannon–Wiener index (H′) and Simpson Index (D) peaked in the 32–36 mm zone and were lowest in the 28–32 mm zone, suggesting that moderate precipitation supports higher species diversity. The H′ was ranked as follows: 0.66 (32–36 mm) > 0.59 (>36 mm <25 mm) > 0.45 (25–28 mm) > 0.35 (28–32 mm). The D was ranked as follows: 0.71 (32–36 mm<25 mm) > 0.69 (>36 mm) > 0.52 (25–28 mm) > 0.40 (28–32 mm). Pielou’s evenness (J) was highest in the areas with <25 mm and lowest in the 28–32 mm zone, likely due to the dominance of a few species: J = 0.86 (<25 mm) > 0.68 (>36 mm) > 0.62 (32–36 mm) > 0.50 (25–28 mm) > 0.47 (28–32 mm). The community cover was highest in the >36 mm zone (0.57), indicating that adequate moisture significantly enhances biomass accumulation.
- M zone: Vegetation was mainly found in the 25–28 mm, 28–32 mm, and >36 mm zones. The H′, J, and cover all peaked in the 28–32 mm zone, indicating optimal diversity conditions under moderate precipitation and slope. The rankings were as follows: H′ = 0.81 (28–32 mm) > 0.49 (25–28 mm) > 0.44 (>36 mm); D = 0.69 (25–28 mm) > 0.49 (>36 mm) > 0.17 (28–32 mm); J = 0.84 (28–32 mm) > 0.57 (25–28 mm = >36 mm); and cover = 0.79 (28–32 mm) > 0.57 (25–28 mm) > 0.51 (>36 mm).
- H zone: Vegetation was sparse and recorded only in the 28–32 mm zone. Despite the limited number of plots, this area showed relatively high vegetation cover and balanced community structure. The H′ and J were both moderate to high, indicating that even under a generally low ecological capacity, suitable local hydrothermal and slope conditions can support a certain level of biodiversity.

### 3.3. Analysis of the Influencing Mechanism of Biodiversity

#### 3.3.1. Model Construction and Measurement Model Evaluation

**Figure 6:**
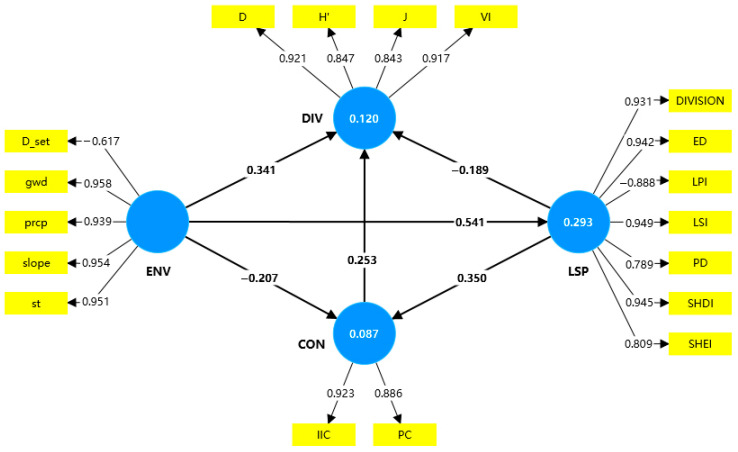
Impact pathways of biodiversity based on PSE-SEM.

Based on the PLS-SEM conceptual framework (Figure 6), we used SmartPLS 4.0 to analyze the causal pathways among the environmental factors, landscape patterns, landscape connectivity, and plant biodiversity in the Hotan River Basin.

All the latent variables demonstrated adequate reliability and validity. Cronbach’s alpha values exceeded 0.70 (range: 0.774–0.920), and the composite reliability (C.R.) values were above 0.90, indicating strong internal consistency. All the standardized factor loadings exceeded 0.70, except for D_set, and the average variance extracted (AVE) values were above 0.70, confirming good convergent validity (Table 6). The D_set was retained because its negative loading direction was theoretically consistent with a human-disturbance gradient in arid oases, its absolute loading (0.617) remained acceptable for an ecologically essential indicator, and its inclusion preserved construct coverage without compromising the reliability or convergent validity.

The discriminant validity was verified by the Fornell–Larcker criterion, where the square roots of AVE values exceeded the inter-construct correlations (Table 7). In addition, the Heterotrait–Monotrait (HTMT) ratios for all the variable pairs fell below the critical threshold (1.0), indicating clear construct separation (Table 6).

#### 3.3.2. Structural Model Fit and Path Coefficient Analysis

The structural model showed a good fit with an SRMR value of 0.046 (<0.1). Bootstrapping with 5000 resamples revealed several statistically significant paths (Table 8):

- H1: The environmental factors had a positive effect on biodiversity (β = 0.341, *p* < 0.001; 95% CI excludes 0)—supported.
- H2: The environmental factors positively affected the landscape patterns (β = 0.541, *p* < 0.001; 95% CI excludes 0)—supported.
- H3: The effect of environmental factors on connectivity was non-significant (β = −0.207; 95% CI includes 0)—not supported.
- H4: The landscape pattern negatively influenced biodiversity (β = −0.189, *p* < 0.05; 95% CI excludes 0)—supported.
- H5: The landscape pattern positively enhanced connectivity (β = 0.350, *p* < 0.001; 95% CI excludes 0)—supported.
- H6: The connectivity positively promoted biodiversity (β = 0.253, *p* < 0.05; 95% CI excludes 0)—supported.

The model demonstrated strong explanatory power, with R^2^ values of 0.120 for biodiversity, 0.087 for connectivity, and 0.293 for landscape pattern.

The f^2^ values for DIV, CON, and LSP were 0.066, 0.095, and 0.414, respectively. According to Cohen’s criteria, the ENV exerted a large effect on the LSP (f^2^ = 0.414), while the relationships among the other latent variables were relatively weak. The predictive relevance was verified using blindfolding (omission distance = 7). All the Q^2^ values were greater than zero (Table 9), indicating adequate predictive accuracy for the key latent variables.

#### 3.3.3. Mediation Effects

To examine the mediating mechanisms among the environmental factors, landscape structure, connectivity, and biodiversity, five indirect pathways were tested (Table 10): H7 (ENV → CON → DIV), H8 (LSP → CON → DIV), H9 (ENV → LSP → DIV), H10 (ENV → LSP → CON), and H11 (ENV → LSP → CON → DIV).

The results showed that H7 was not supported: the 95% bootstrap confidence interval for the indirect effect crossed zero, indicating no mediating role of connectivity in the ENV → DIV pathway. In contrast, H8–H11 were statistically significant (*p* < 0.001) with confidence intervals that did not include zero:

- H8 confirmed that connectivity mediates the relationship between landscape pattern and biodiversity.
- H9 indicated a negative mediation effect, where the landscape pattern transmits the indirect influence of the environmental factors on biodiversity.
- H10 supported a full mediation, where the landscape pattern entirely mediates the relationship between the environmental factors and connectivity.
- H11 revealed a chain mediation effect, suggesting that the environmental factors influenced biodiversity indirectly via sequential effects on the landscape pattern and connectivity.

#### 3.3.4. Path Effect Analysis

To comprehensively understand the mechanisms underlying the interactions among the variables, the direct, indirect, and total effects of the structural model were analyzed (Table 11). The results revealed the following:

- Because the ENV → CON path was not significant, connectivity did not mediate the ENV → DIV relationship; along this route, the environmental factors influenced diversity only directly. By contrast, in the sequential path ENV → LSP → CON, the landscape pattern fully mediated the link between the environmental factors and connectivity.
- For the LSP → CON → DIV pathway, the landscape pattern exerted a negative direct effect on diversity (β = −0.189, *p* < 0.05) and a positive indirect effect via connectivity (β = 0.089, *p* < 0.01), yielding a competitive mediation with a small negative total effect (total = −0.100).
- Along ENV → LSP → DIV, the direct effect was positive (β = 0.341, *p* < 0.001), whereas the indirect effect via the landscape pattern was negative (β = −0.103, *p* < 0.05), indicating competitive mediation with a net positive total effect (total = 0.238).
- A chained mediation from the ENV through LSP and CON to DIV was significant (β = 0.389), underscoring a multilevel mechanism whereby the environmental drivers reshape the landscape structure, which in turn modulates connectivity and ultimately regulates plant diversity in drylands.

## 4. Discussion

### 4.1. Landscape Area Dynamics and Conversion Pathways

At the basin scale, land-use/cover change in arid river systems reflects coupled hydroclimatic and anthropogenic influences on landscape composition [8]. In the Hotan River Basin (2000–2023), bare land remained the dominant class (~83% of area) but exhibited a consistent net decline, with most conversions directed to shrubland and, secondarily, cultivated land. This shift mirrors the patterns reported for desert–oasis ecotones across the Tarim Basin, where modest improvements in water availability and local water management have facilitated vegetative infilling on formerly barren substrates [49]. Construction land, although a small fraction of the total, expanded rapidly—largely at the expense of shrubland—consistent with peri-urban growth and settlement infill along oasis margins and transport corridors [50]. Together, these trajectories indicate a dual-driver regime: the hydrologically enabled “greening” of bare surfaces alongside the steady encroachment of built-up areas into ecologically functional spaces.

The forward simulations reinforce these tendencies while clarifying pathway differences. Under SSP1-2.6, bare land decreases and both shrubland and cultivated land increase by 2035 and 2050, whereas construction land expands in all the scenarios, most prominently under SSP5-8.5. SSP2-4.5 yields mixed responses in the vegetated classes but maintains the construction land’s growth. Read against the historical baseline, these projections imply a continued net conversion from bare land to vegetated types, with the rate and spatial footprint contingent on the socioeconomic trajectory and water availability. In short, the basin’s landscape is likely to remain dominated by bare land and shrubland through mid-century, with the persistent expansion of construction land—a combination that will keep the composition of the desert–oasis mosaic tightly governed by both climate-sensitive hydrology and human development.

### 4.2. Vegetation Distribution and Hydrothermal Adaptation

Our study reveals that the vegetation distribution in the study area shows strong alignment with the environmental gradients, especially precipitation and slope. We reveal that the areas with moderate slopes and annual precipitation between 25 and 36 mm exhibit the highest diversity indices (H′ and D) and relatively stable community structures. This finding supports the ecological niche theory in arid zones, where species richness peaks under intermediate energy and stress conditions [26,27,51]. The dominant species, such as *Populus euphratica* and *Tamarix chinensis,* demonstrate high adaptability to steep slopes and low water availability, forming critical transitional communities between riparian zones and deserts [11,32].

The composition and diversity of plant communities are influenced by the combined effects of slope and precipitation. The lower and mid-slope zones (L and M) support higher species richness, including regional endemics such as *Calligonum roborowskii* and *Seriphidium korovinii*, which are sensitive indicators of desertification. In contrast, the high-slope zones (H) exhibit limited species richness due to the constraints imposed by both moisture availability and the topographic conditions. These patterns align with broader desert ecosystem studies from Central Asia, affirming the role of slope–precipitation interactions in shaping biodiversity [14,27,33].

### 4.3. Pathway Mechanisms Linking Landscape Pattern, Connectivity, and Biodiversity

#### 4.3.1. Direct and Indirect Effects of Landscape Pattern on Biodiversity

The spatiotemporal configuration of landscapes is a major component and driver of global change, with pervasive consequences for ecosystem services and biodiversity. In our PLS-SEM, the landscape pattern (LSP) exerts both a direct negative effect on plant diversity (β_direct = −0.189, *p* < 0.05) and an additional negative indirect effect via connectivity (β_indirect = −0.103, *p* < 0.05). These pathways are consistent with the 2000–2023 observations of stronger fragmentation and weaker aggregation in the basin (NP/PD and SHDI/SHEI ↑; AI/CONTAG ↓; IJI and DIVISION ↑; see Section 3.1.3).

Ecologically, the LSP indicators used here capture distinct mechanisms. The ED and LSI tend to intensify the edge effects under hot, high-resistance matrices, which lowers the J and raises the D, and also suppresses the functional connectivity (IIC/PC). The PD and DIVISION quantify fragmentation and isolation; increases in them typically reduce dispersal and rescue effects, thereby depressing the H′ and J [33,52]. The LPI reflects the control of the dominant matrix. In the Hotan River Basin—where bare land retains the largest patch—the high LPI implies a resistance-dominated matrix that disrupts path continuity and accessibility, lowering the IIC/PC and diversity metrics; the converse can occur if the largest patch is a continuous shrubland corridor [20,41]. Finally, SHDI/SHEI indicate compositional heterogeneity and balance. When fragmentation remains below the threshold, a higher SHDI/SHEI can increase microhabitat variety and support a higher H′; however, if rising SHDI/SHEI co-occurs with CONTAG decline and PD/DIVISION increase, heterogeneity shifts toward a finely “speckled” mosaic that undermines connectivity and community stability [53].

Together, these processes explain the net negative direct effect of LSP on diversity in an arid, high-resistance context: elevated ED/LSI/PD/DIVISION and a persistently high bare land LPI outweigh any benefits of moderate heterogeneity. Because the LSP also depresses connectivity, and connectivity in turn promotes diversity (Section 4.3.2), the LSP → CON → DIV route contributes an additional negative (competitive) mediation. These findings align with the circuit-theory arguments that functional connectivity, not mere structural adjacency, governs ecological exchange in fragmented drylands, and with the threshold responses to fragmentation reported for arid systems [52].

#### 4.3.2. Connectivity as the Bridge from Structure to Biodiversity

The connectivity (CON) showed a significant positive direct effect on diversity (β = 0.242, *p* < 0.001), whereas ENV → CON was non-significant, making LSP → CON → DIV the principal indirect pathway. The IIC and PC increased with an assumed dispersal distance and plateaued near ~10 km, indicating a functional threshold: below this scale, the network remains partly broken; above it, most vegetated patches become functionally linked. This threshold accords with the field conditions—wind and seasonal flows are the dominant vectors—and explains why local expansions of built-up land or extensive bare land matrices (“hard edges–high resistance–breaks”) markedly reduce the effective path probabilities and increase travel times [40]. Empirical and modeling studies similarly show that once the IIC/PC cross a scale-dependent threshold, colonization/gene flow accelerate and communities stabilize; below it, systems drift toward isolation and degradation [20]. Thus, raising the IIC/PC is not a mere numeric gain; they represent lower dispersal costs, more permeable corridors, and higher renewal probabilities—the proximate mechanisms behind the positive CON → DIV effect.

The observed coupling between the LSP and CON is also clear: a higher PD/DIVISION with a lower AI/CONTAG suppresses the IIC/PC, whereas linear or stepping-stone ribbons of shrubland/wetland along riparian corridors (moderate ED/LSI, higher CONTAG) enhance functional linkages. Given that ENV → CON was not significant, the basin’s functional network is generated through a staged process—first reconfiguring the structure (LSP), then expressing connectivity (CON). Consequently, the rising SHDI/SHEI between 2000 and 2023 does not automatically translate into diversity gains when the bare land LPI remains high and the PD/DIVISION increase, unless the ~10 km functional threshold is met. Connectivity, therefore, is the necessary bridge that converts “pattern optimization” into sustained biodiversity benefits.

#### 4.3.3. Hierarchical Roles of Environmental Factors

The environmental factors (ENV) had significant positive direct effects on both the diversity and landscape pattern (ENV → DIV: β = 0.341; ENV → LSP: β = 0.541; both *p* < 0.001), but no direct effect on the connectivity (ENV → CON: β = −0.207, 95% CI includes 0). This implies a staged “environment → structure → function → diversity” process: the environmental constraints first reshape the patch geometry and composition (LSP), which then alter the dispersal costs and accessibility (CON), ultimately influencing community diversity (DIV) [20,54,55].

The ENV latent variable comprised the D_set (negative loading indicating human disturbance), gwd, prcp, slope, and st. The disturbance gradient matches the spatial signal of a rising PD/LSI/IJI and a declining CONTAG (Section 3.1.3). The gwd directly constrains *Populus*/*Tamarix* persistence; deeper water tables contract shrubland patches and depress SHDI/SHEI, narrowing riparian corridors and lowering the IIC/PC and the H′/J [51,56]. Water availability is the primary limitation in drylands [49]; in our plots, the H′ and D peaked at ~32–36 mm annual precipitation (Section 3.2), consistent with enhanced coexistence and rescue effects at moderate moisture, and with concomitant gains in SHDI/SHEI. The slope captures microtopographic control of soil moisture, radiation, and substrate stability; steeper slopes lengthen edges (ED) and increase shape complexity (LSI), raise the PD/ED and reduce the IIC/PC once the thresholds are exceeded, and lower the establishment probabilities (H′/J down, D up) [57]. The observed advantage of low–mid slopes for cover and diversity (Section 3.2) corroborates these mechanisms. The soil type governs water holding and nutrients, often sustaining a high bare land LPI, lower CONTAG, and higher DIVISION, which depress the IIC/PC and shifts communities toward a lower H′/J and higher D [54].

In sum, the positive ENV → DIV pathway reflects the direct benefits of moisture and favorable topo-edaphic conditions; the strong ENV → LSP effect indicates that the environment first reconfigures the spatial “skeleton”; the lack of a direct ENV → CON link suggests that connectivity improvements depend on prior structural modification in this basin.

#### 4.3.4. An Integrated Chain Mechanism and Its Consistency with Prior Work

Collectively, the model supports a four-level chain: ENV → LSP → CON → DIV. The LSP fully mediates ENV → CON, and DIV is co-determined by the direct environmental signal (β = 0.341), structural constraint from the LSP (β = −0.189), and functional promotion by CON (β = 0.253), plus multiple indirect paths. The chained mediation (ENV → LSP → CON → DIV) has the largest total effect (0.389), indicating that in arid mosaics, environmental constraints typically act first through spatial reconfiguration and then through functional linkage to shape biodiversity.

These findings are consistent with large-scale syntheses in which climate/water set broad species distributions, whereas landscape-level heterogeneity and connectivity exert stronger control on local diversity [49,58,59]. They also echo cross-regional comparisons (e.g., Frishkoff [60]; Beissinger [61]) while underscoring a dryland specificity: under high-resistance matrices—reflected by the high bare land LPI—and concurrent human disturbance, functional connectivity becomes the key buffer against climatic stress. Critically, only when networks surpass the ~10 km dispersal threshold and structural resistance is reduced can increases in SHDI/SHEI yield net biodiversity gains rather than fragmentation costs.

### 4.4. Theoretical and Methodological Contributions

Anchored in the environmental sustainability framework of Ribeiro et al. [62], this study adopts a scale–place–space perspective and embeds dryland biodiversity into a causal chain of environment → structure → function → diversity, yielding a transferable analytical framework for arid social–ecological systems. At the scale dimension, macro-level hydroclimate and human disturbance are downscaled into meso-level landscape metrics (PD, ED, LSI, LPI, SHDI, SHEI, DIVISION) and micro-level functional connectivity (PC, IIC), revealing an ~10 km dispersal threshold that links landscape resistance to realized community diversity. At the place dimension, the groundwater depth, soil type, and proximity to settlements are explicitly incorporated for the oasis–desert context, showing that compositional heterogeneity translates into biodiversity gains only when fragmentation remains sub-threshold and matrix resistance is reduced. At the space dimension, remotely sensed configuration change is coupled with movement-related functional processes and the SSP1-2.6/SSP2-4.5/SSP5-8.5 scenarios, converting “pattern change” into an operational process diagnosis. Overall, the work clarifies how scale interacts with geographic context, extends sustainability theory to arid inland river basins, and provides a reusable indicator set and framework to inform biodiversity conservation and landscape governance.

Methodologically, this study advances landscape–ecological modeling in two complementary ways. First, it integrates environmental (ENV), structural (LSP), and functional (CON) components within a PLS-SEM architecture, thereby identifying the multiple indirect and chain-mediated pathways among the drivers and biodiversity responses. Multi-source data—multi-temporal satellite imagery, DEM-derived terrain, climate records, and 57 field plots—are harmonized on a 4 × 4 km^2^ grid to ensure cross-dataset comparability. Pairing classic landscape indices (e.g., SHDI, PD, CONTAG) with Conefor-based connectivity metrics (PC, IIC) enables a comprehensive assessment of structure–function–diversity linkages and moves beyond a metric description toward mechanism-oriented inference. Second, we couple PLUS with a causal framework to bridge “what-is” and “what-if.” The PLUS model combines machine learning suitability (LEAS/Random Forest) with patch-level CA dynamics (CARS), allowing us to (i) explain the land-use drivers and simulate patch evolution consistent with recent applications in China’s drylands, and (ii) generate scenario outputs (SSP1-2.6/SSP2-4.5/SSP5-8.5; 2035/2050) that are directly interpretable within the ENV → LSP → CON → DIV chain. In practice, PLS-SEM quantifies how environmental constraints propagate through spatial configurations and functional connectivity to affect diversity, while PLUS projects the spatial trajectories of those constraints under alternative futures. Together, they deliver a transferable, scale-aware template that links diagnosis (causal pathways) with prognosis (scenario-based maps), thereby positioning our findings squarely within contemporary environmental—sustainability research and supplying actionable evidence for dryland river-basin planning.

### 4.5. Implications and Limitations

#### 4.5.1. Implications for Sustainable Landscape Planning

Our findings translate the “environment → structure → function → diversity” chain into four actionable principles for arid river basins. (i) Connectivity-first design: Because biodiversity increased with the functional connectivity and an ~10 km dispersal threshold emerged from the IIC/PC analysis, planning should prioritize continuous riparian and shrubland corridors and maintain stepping-stone patches at ≤10 km intervals, while minimizing the breaks created by extensive bare land or new construction. (ii) Manage fragmentation, not just heterogeneity: The instances where SHDI/SHEI rose alongside a higher PD/DIVISION and lower AI/CONTAG show that more heterogeneous mosaics can still undermine habitat function; management targets should therefore include reducing the bare land LPI, stabilizing the ED/LSI at moderate levels, and removing corridor pinch points. (iii) Place-based water–land coordination: Plots in zones with shallower groundwater and moderate precipitation supported higher diversity; maintaining environmental flows and safeguarding shallow-groundwater belts can widen riparian corridors and raise the PC/IIC without large land conversions. (iv) Scenario-informed zoning: The SSP projections indicate a persistent expansion of construction land; guiding this growth away from high-centrality links in the connectivity network and toward low-resistance areas could preserve basin-scale permeability under climate variability.

#### 4.5.2. Limitations and Future Directions

First, the sample size (n = 57) may limit the model’s robustness. Future work should expand the spatial coverage using UAV high-resolution imagery and lightweight object-detection models (e.g., YOLOv10) [63] coupled with Sentinel-2 data [64] to automatically delineate vegetation patches and high-density species areas, and cross-validate against field plots to reduce sampling bias. Second, several socio-ecological covariates (e.g., population density, distance to roads) were excluded due to low measurement loadings (<0.5), potentially omitting environment–biodiversity pathways. Higher-resolution, process-proximal predictors (e.g., grazing intensity, groundwater extraction), together with variable transformations, hierarchical modeling, and multi-model sensitivity analyses should be used to test the robustness of human-pressure effects on the structure and connectivity. Lastly, our biodiversity metrics emphasize taxonomic diversity; integrating functional traits and phylogenetic diversity, ideally within a long-term monitoring design, will better capture ecosystem stability and adaptive capacity under climate stress.

## 5. Conclusions

Guided by environmental sustainability theory and a scale–place–space perspective, this study employed multi-source data integration and PLS-SEM modeling to investigate the interactions among environmental factors, landscape structure, connectivity, and plant biodiversity in the Hotan River Basin. The main conclusions are as follows:

- Landscape Pattern Transformation: Between 2000 and 2023, the bare land declined, converted mainly into shrubland and cultivated land, while the construction land expanded. The NP/PD, SHDI/SHEI, and IJI/DIVISION rose, and the AI/CONTAG fell—indicating finer, more heterogeneous mosaics in the study area. The projections (SSP1-2.6/SSP2-4.5/SSP5-8.5) to 2035/2050 retained bare land + shrubland dominance, with construction land increasing in all the scenarios (strongest under SSP5-8.5) and the vegetation responses varying by pathway.
- Plant Community Composition and Diversity Drivers: The spatial distribution of plant community composition and diversity was jointly influenced by precipitation and slope gradients. Areas with low-to-moderate slopes exhibited higher species richness, evenness, and vegetation cover, with diversity patterns varying along the precipitation gradients. Regional endemics from the Kunlun–Tarim ecotone were more prevalent in the wetter zones, underscoring the ecological importance of these habitats for dryland biodiversity conservation.
- Mechanisms of Biodiversity Regulation via PLS-SEM Modeling: The PLS-SEM captured the multifactorial controls on plant diversity, with strong model fit and explanatory power. The landscape structure exerted both direct effects on biodiversity and indirect effects mediated by connectivity. The environmental factors showed competitive mediation—positive direct effects on diversity but negative indirect effects via landscape structure. The chained pathway (ENV → LSP → CON → DIV) was the dominant indirect route, indicating that in drylands environmental constraints first reconfigure spatial patterns, which then propagate to community responses through functional connectivity, underscoring the central role of connectivity in buffering climatic stress and sustaining biodiversity.
- Contributions, Limitations, and Future Directions: Grounded in a scale–place–space perspective, this study embeds dryland biodiversity within an environment → structure → function → diversity chain, yielding connectivity-first and fragmentation-aware guidance for arid-basin planning. The limitations include incomplete spatial coverage and sparse socio-ecological covariates, which may constrain inferences. Next, we will integrate UAV high-resolution imagery, Sentinel-2, and lightweight detectors (e.g., YOLOv10) for automated patch mapping and plot validation; add human-pressure indicators and process-proximal predictors with hierarchical and sensitivity analyses; and extend beyond the taxonomic metrics to functional/phylogenetic diversity within long-term monitoring.

## Figures and Tables

**Table 1:** Data sources and description.

Data Type	Years	Resolution/Scale	Source	Application
Landsat imagery (TM/ETM+/OLI)	2000, 2012, 2023	30 m	USGS Earth Explorer	Supervised classification of landscape types
DEM	2023	30 m	Geospatial Data Cloud	Elevation, slope calculation
National vegetation map	2012, 2023	1:1,000,000	Peking University	Auxiliary verification of vegetation distribution/community structure
Roads, rivers, settlements, administrative boundaries	2020	Vector	NCSGI (webmap.cn)	Drivers for PLUS and exogenous variables for PLS-SEM
Temperature, precipitation, population density, GDP, soil types	2023	1 km × 1 km	RESDC (resdc.cn)	Drivers for PLUS; environmental predictors (PLS-SEM)
LUH2 land-use scenarios (SSP126, SSP245, SSP585)	2035, 2050	1 km × 1 km	LUH2 (luh.umd.edu)	Inputs for PLUS simulations under three SSP scenarios

**Table 2:** Landscape connectivity value of brushland under partial distance thresholds.

	400 m	1 km	2 km	4 km	8 km	10 km	16 km	20 km
IIC	1160.960	1169.802	1287.958	1343.202	1357.357	1364.034	1364.034	1364.034
PC	1494.676	1615.565	1740.949	1841.352	1909.075	1924.908	1947.691	1959.936

**Table 3:** Landscape pattern index of Hotan River Basin in 2000, 2012, and 2023.

Years	NP	AI	SHDI	SHEI	CONTAG	IJI	DIVISION
2000	58,687	99.6539	0.4888	0.2728	85.9054	34.4709	0.2492
2012	65,656	99.6018	0.4977	0.2778	85.6009	36.1645	0.2505
2023	68,817	99.5765	0.5022	0.2803	85.4525	39.1782	0.2508

**Table 4:** Species composition and IV at different slopes.

Family	Genus	Species	IV		
			0–4° (L)	4–11° (M)	11–71° (H)
Salicaceae	*Populus*	*Populus pruinosa*	0.34 ± 0.06	0.13 ± 0.01	-
		*Populus euphratica*	0.31 ± 0.07	0.33 ± 0.06	0.35 ± 0.11
Tamaricaceae	*Tamarix*	*Tamarix chinensis*	0.15 ± 0.04	0.25 ± 0.02	0.20 ± 0.10
	*Reaumuria*	*Reaumuria soongorica*	0.52 ± 0.23	0.12 ± 0.08	-
Solanaceae	*Lycium*	*Lycium ruthenicum*	0.19 ± 0.08	0.13 ± 0.10	0.05 ± 0.04
Poaceae	*Phragmites*	*Phragmites australis*	0.35 ± 0.08	-	-
Fabaceae	*Glycyrrhiza*	*Glycyrrhiza inflata*	0.06 ± 0.02	0.01 ± 0.01	
	*Alhagi*	*Alhagi sparsifolia*	0.17 ± 0.08	-	0.11 ± 0.09
	*Halimodendron*	*Halimodendron halodendron*	0.09 ± 0.09	0.01 ± 0.01	-
	*Sophora*	*Sophora alopecuroides*	0.12 ± 0.10	-	-
Chenopodiaceae	*Halostachys*	*Halostachys caspica*	0.05 ± 0.05	0.01 ± 0.01	-
	*Halogeton*	*Halogeton glomeratus*	-	0.03 ± 0.01	-
	*Bassia*	*Bassia dasyphylla*	-	0.16 ± 0.01	-
	*Sympegma*	*Sympegma regelii*	-	0.30 ± 0.01	-
Asteraceae	*Karelinia*	*Karelinia caspica*	0.09 ± 0.06	0.06 ± 0.01	-
	*Seriphidium*	*Seriphidium korovinii*	0.41 ± 0.04	-	-
Asclepiadaceae	*Cynanchum*	*Cynanchum chinense*	0.11 ± 0.06	-	-
Polygonaceae	*Calligonum*	*Calligonum roborowskii*	-	0.27 ± 0.01	-
Zygophyllaceae	*Zygophyllum*	*Zygophyllum rosovii*	-	0.14 ± 0.01	-
Apocynaceae	*Poacynum*	*Poacynum hendersonii*	0.08 ± 0.04	0.03 ± 0.02	-
Amaranthaceae	*Kalidium*	*Kalidium foliatum*	0.03 ± 0.02	0.02 ± 0.01	-

**Table 5:** Characteristics of vegetation in areas with different slopes and precipitation distributions.

Slope	Precipitation	Cover	Shannon–Wiener (H′)	Pielou (J)	Simpon (D)
0–4°(L)	<25 mm	0.16 ± 0.09	0.59 ± 0.10	0.86 ± 0.04	0.71 ± 0.08
	25–28 mm	0.54 ± 0.07	0.45 ± 0.08	0.50 ± 0.08	0.52 ± 0.08
	28–32 mm	0.06 ± 0.01	0.35 ± 0.13	0.47 ± 0.17	0.40 ± 0.16
	32–36 mm	0.48 ± 0.06	0.66 ± 0.14	0.62 ± 0.10	0.71 ± 0.14
	>36 mm	0.57 ± 0.01	0.59 ± 0.08	0.68 ± 0.07	0.69 ± 0.01
4–11°(M)	25–28 mm	0.57 ± 0.09	0.49 ± 0.08	0.57 ± 0.08	0.65 ± 0.12
	28–32 mm	0.79 ± 0.07	0.81 ± 0.01	0.84 ± 0.04	0.17 ± 0.07
	>36 mm	0.51 ± 0.02	0.44 ± 0.19	0.57 ± 0.03	0.49 ± 0.10
11–71°(H)	28–32 mm	0.74 ± 0.01	0.53 ± 0.10	0.60 ± 0.08	0.38 ± 0.05

**Table 6:** Reliability and validity indicators of the model.

Latent Variables	Observed Variables	Factors Loading	AVE	C.R.	Cronbach’s Alpha	Confidence Interval of HTMT
Biodiversity (DIV)	VI	0.917	0.779	0.934	0.920	Excluding 1
	D	0.921				
	H′	0.847				
	J	0.843				
Connectivity (CON)	IIC	0.923	0.818	0.900	0.780	Excluding 1
	PC	0.886				
Environment (ENV)	D_set	−0.617	0.792	0.909	0.810	Excluding 1
	gwd	0.958				
	st	0.951				
	slope	0.954				
Landscape (LSP)	ED	0.942	0.802	0.935	0.774	Excluding 1
	PD	0.789				
	LSI	0.949				
	LPI	−0.888				
	DIVISION	0.931				
	SHDI	0.945				
	SHEI	0.809				

**Table 7:** The square root of the potential factor AVE value.

	DIV	CON	ENV	LSP
DIV	0.883 ^1^			
CON	0.202	0.905		
ENV	0.234	−0.018	0.890	
LSP	0.055	0.238	0.541	0.896

^1^ The values on the diagonal are the square root of the AVE.

**Table 8:** Path coefficient testing of direct effect.

Pathway	Coefficient (β)	Std. Deviation	*t*-Statistic	*p*-Value	95% Confidence Interval
ENV -> DIV	0.341	0.067	5.125	0.000	[0.196, 0.461]
ENV -> LSP	0.541	0.048	11.194	0.000	[0.439, 0.625]
ENV -> CON	−0.207	0.096	2.158	0.031	[−0.361, 0.018]
LSP -> DIV	−0.189	0.078	2.434	0.015	[−0.342, −0.036]
LSP -> CON	0.350	0.072	4.839	0.000	[0.187, 0.473]
CON -> DIV	0.253	0.075	3.356	0.001	[0.133, 0.424]

**Table 9:** Q^2^ of latent variables.

	SSO	SSE	Q^2^ (=1 − SSE/SSO)	f^2^
DIV	228	213.730	0.063	ENV -> DIV: 0.091; Con -> DIV: 0.066; LSP -> DIV: 0.026
CON	114	110.470	0.031	LSP -> CON: 0.095
LSP	342	251.823	0.264	ENV -> LSP: 0.414

**Table 10:** Path coefficient testing of indirect effect.

Pathway	Coefficient (β)	Std. Deviation	*t*-Statistic	*p*-Value	95% Confidence Interval
ENV → CON → DIV	−0.052	0.026	1.983	0.047	[−0.090, 0.007]
LSP → CON → DIV	0.089	0.027	3.338	0.001	[0.042, 0.145]
ENV → LSP → DIV	−0.103	0.042	2.439	0.015	[−0.187, −0.020]
ENV → LSP → CON	0.190	0.044	4.344	0.002	[0.099, 0.273]
ENV → LSP → CON → DIV	0.048	0.016	3.036	0.002	[0.022, 0.083]

**Table 11:** Direct, indirect, and total effects of structural paths.

Pathway	Direct Effect	Indirect Effect	Total Effect
ENV -> CON -> DIV	0.341 ***	−0.052	-
LSP -> CON -> DIV	−0.189 *	0.089 **	−0.100
ENV -> LSP -> DIV	0.341 ***	−0.103 *	0.238
ENV -> LSP -> CON	−0.207	0.190 ***	-
ENV -> LSP -> CON -> DIV	0.341 ***	0.048 **	0.389

* *p* < 0.05, ** *p* < 0.01, *** *p* < 0.001.

## Data Availability

The data presented in this study are available on request from the author; the data are also part of an ongoing study.

## Abbreviations

The following abbreviations are used in this manuscript:

PLUS	Patch-generating Land Use Simulation Model
PLS-SEM	Partial Least Squares Structural Equation Modeling
PD	Patch Density
AI	Aggregation Index
SHDI	Shannon’s Diversity Index
SHEI	Shannon’s Evenness Index
CONTAG	Contagion Index
IJI	Interspersion and Juxtaposition Index
DIVISION	Landscape Division Index
NP	Number of Patches
LPI	Largest Patch Index
LSI	Landscape Shape Index
ED	Edge Density
SIDI	Simpson’s Diversity Index
SIEI	Simpson’s Evenness Index
IIC	Integral Index of Connectivity
PC	Probability of Connectivity
IV	Importance Value
D	Simpson’s Dominance Index
H′	Shannon–Wiener Diversity Index
J	Pielou’s Evenness Index
ENV	Environmental Factors
LSP	Landscape Pattern
CON	Connectivity
DIV	Plant Diversity
